# The British Medical Association at Exeter

**Published:** 1907-08-03

**Authors:** 


### The British Medical Association at Exeter.

BY OUR SPECIAL REPORTERS.

Exeter, Sunday, July 28.

The seventy-fifth annual meeting of the British Medical Association promises to be a success—so far at least as a good attendance of members and a vigorous display of interest on the part of those concerned in the meetings go. The regular work of the conference will not begin till Wednesday, July 31, and as at present anticipated the proceedings will terminate on Friday afternoon. The programme which has been arranged is a lengthy one, and one, moreover, that promises well. It is a programme, however, the successful execution of which demands a smoothness and precision of organisation and a dexterity of management which call for great sacrifices on the part of the officers of the various sections and equally great energy on the part of the general staff.

### Exeter: The Place and Its People.

There is something specially attractive for an army of materialistic professional men in an old cathedral town such as Exeter is. Not for that reason, however, do we imagine that Exeter has been selected for this year's annual meeting. It is for the second time that the Association meets here, and there have been great changes since 1872, when the members of the Provincial Medical and Surgical Association, which blossomed forth as the British Medical Association at the Birmingham Conference in 1886, visited the town. A glance at the list of members then and now will convey at once, even to the uninitiated, the significance and importance of the conference that meets here this week. In 1842, under the presidency of the late Mr. John Haddy Jones, surgeon to the Devon and Exeter Hospital, the Association had a membership of less than 200. At the present moment more than 19,000 names are on the members' roll of the Association, and nearly 1,200 medical men, the greater part of them active associates, are taking part in the proceedings of the annual meeting.

Exeter as a meeting-place has been well chosen. It is the centre of some of the most beautiful country in England; a veritable host of attractions lie at hand for the tourist. In fact, it is possible that members will not find the sectional meetings so interesting as they might have proved amidst surroundings less potent to charm forth the newcomer into voyages of exploration, discovery, and appreciation. To start with, there is the cathedral, around which the Chancellor "has kindly consented to conduct parties." The ex-President of the Devon and Exeter Archæological Society will take parties round the city to see the Guildhall, Tuckers' Hall, the Elizabethan home of the Guild of Merchant Adventurers, the Hall of the College of Vicars Choral, founded in 1388 by Bishop Brantyngham, old Exeter houses, and old Exeter churches.

### The Railways.

The two great railway lines that serve Exeter have made convenient arrangements for the benefit of members and their friends. The London and South-Western Railway, whose Waterloo-Exeter service of trains is all that the visiting associate can desire, has arranged a series of daily excursions to Bideford, Westward Ho, Clovelly, Ilfracombe, Lynton, Woody Bay, Watersmeet, Seaton, Exmouth, Ottery St. Mary, and Sidmouth. The Great Western, whose lines serve the picturesque Dartmoor and Exmoor districts, has an equally long list of attractions, and the visitor who has little time at his disposal will be rather hard pressed to decide between them all. Full particulars of these excursions are obtainable at the Albert Rooms, where the Association headquarters are.

### The Guide-Book.

A word of cordial praise for the customary local guide-book. This is "A Book of the South-West," beautifully illustrated by more than 200 photographs, and containing, besides much useful information concerning the climatology, sport, and local clubs, two interesting articles which deserve a wider publicity than they seem likely to obtain. One is Mr. Quiller Couch's fine essay on Cornwall, and the other is Mr. Alfred Wallis's note on the history of Cornwall and Devon.

### The Local Clubs.

The people of Exeter have not been halting in their hospitality. They have put everything at the disposal of their guests. The town is full, and lodgings are practically unobtainable, unless previously arranged for. The local golf and social clubs have extended their hospitality to members of the Association.

### Business Done.

On Saturday the annual general meeting took place, followed immediately afterwards by the Representative meeting. The latter was continued on Monday, but press representatives, even those of the medical press, are not admitted, and the agenda is marked "Private and Confidential." Sunday was devoted to sight-seeing and attendance at the local churches, where, however, no special services were held. This seems a strange omission on the part of the clergy.

### Why Shut Out the Press?

On Tuesday the Association began its sectional work in earnest. The "adjourned representative meeting" was still being held, but as only "representatives" and not even ordinary members, much less press representatives, whether lay or professional, were admitted to its deliberations, it is impossible to state in detail what was achieved by its members. It is a pity that there should be so large an element of secrecy about the representative meetings; "in camera" and "confidential" writ large are not attractive, least so in scientific gatherings, and the attitude of some of the officials at times raises a smile. Even the sectional meetings, at which, presumably, purely professional business, not of a private nature, is discussed, are closed to the press. For an association such as this, which assumes, by its title and by its claims, the pretension of being not only a national but an Imperial organisation, this foolish dread of publicity is absurd. Moreover, it is prejudicial to the best interests of the Association. There are many general practitioners who are not members of the Association, and who cannot therefore attend the sectional meetings in person; there are others who, though members, do not take so close, selfish, and corporate a view of their communications to the Association as some of the officials seem to do. To the former the benefit of the sectional meetings is largely lost because the discussions are only "officially" reported; to the latter it is a manifest injustice that their papers should not be made to appeal to the profession generally instead of only to the members of the Association, numerous though they may be.

### Monday's Work.

The business done by the general member on Monday may be summarised in a phrase "feeling his feet." It is not easy to "get the hang of the show," as one of the representatives remarked irritably. At the Albert Memorial Rooms, indeed, every attention and courtesy was paid to the visitor. He was furnished with a guide-book which is practically the ideal of what such things should be, and everything was made easy for him. But yesterday there was nothing to see. The sections had not yet begun, and all that the curious could do was to saunter round and map out the town in his mind, marking the various buildings and points where meetings or exhibitions were to be held.

### The Civic Welcome.

In the afternoon members turned up in force at the Guildhall, where they were welcomed by the Mayor (Mr. Alderman Reed) and the Sheriff (Dr. Pickard). Dr. Domville, Vice-President of the South Western branch of the Association, the branch which had invited the members to Exeter, introduced the President of the Association (Dr. A. Reeve, of Toronto), the President-elect (Dr. Davey, of Exeter), and the chairman of the Representative meeting (Dr. MacDonald, of Taunton). Dr. Davy, in the course of a short speech, said that it was the duty of the Government to make scientific research to stop disease. The Government had failed in its duty, and in the absence of public effort, the Association had spent something like £30,000 during the last twenty years in doing work which the Government ought to have done for the protection of life.

In the evening the President-elect gave a dinner party, followed by an At Home at his residence.

### The Cathedral Service.

On Tuesday at noon services were held both in the Cathedral and in the Catholic Church of the Sacred Heart in South Street. Both services were well attended, the majority of the members wearing academic costume.

The service in the Cathedral, though not unusually impressive, was picturesque and striking, the range of colour displayed by the various robes and hoods adding greatly to the decorative effect within the nave. The Mayor and Corporation attended in state, and the general public filled every available seat that was left open after the doctors and their ladies had been accommodated. A procession was formed at the west door, the clergy and choir boys leading the way, and the executive and members of the Association, robed or uniformed, following. The sermon was preached by the Bishop of Exeter, who took as his text the 17th verse of the second chapter of St. Mark's Gospel, and discoursed on the subject of Christianity and medical science. The Bishop had an interesting subject, and he treated it in an interesting manner, contriving to bring in Celsus and Origen, Luke the Physician, Eusebius, and Alexander in order to show that Christianity has ever tolerated medicine and even on occasions treated it with respect.

### The Pathological Museum.

This is now open for inspection, and one's first attempt to endeavour to get a general view of the collection proves that the Association has succeeded, and brilliantly, in forming a really useful pathological museum, the educative value of which it would be hard to overestimate.

The collection is housed in Barnfield Hall, and it is, of course, impossible to attempt here more than a casual survey of the exhibits. These are carefully arranged, displayed, mounted, and labelled to perfection, and the catalogue which is handed to visitors is so conscientiously edited that it is of real service. All the exhibits are loan collections, and some of them are unique. Where so many are worthy of praise, it seems invidious to single out any one for special mention, but we cannot refrain from drawing attention to the beautiful series of exhibits illustrating the latest advances in cancer research, for which Dr. Bashford is responsible, and which are on view through the courtesy of the Imperial Cancer Research Fund. Professor Ehrlich sends two charts and several slides illustrating experimental tumour production, and Mr. Cecil Rowntreee, F.R.C.S., exhibits several very interesting thin sections of mammary cancer. On one of the side tables is to be seen a fine collection illustrating the pathology of Bilharziosis, showing the changes found in the genito-urinary tract and the so-called "Bilharzia cirrhosis of the liver." For the majority of these Professor Ferguson, of Cairo, is responsible, though one of the most interesting specimens (399. Bilharzia in prostate) is lent by Professor St. Clair Symmers, of Belfast. Professor Muir, of Glasgow, has lent some very characteristic specimens illustrating the lesions in cerebro-spinal meningitis of the epidemic type. Mr. Moynihan shows a series of specimens illustrating the pathology of gall-bladder lesions, and the specimens in the parasitology and tropical diseases sections are unusually good. On the ledge devoted to specimens illustrative of diseases of women are to be found Professor Strassmann's loan collection, ideally mounted, and very fully labelled. Dr. William Hunter shows a number of very interesting specimens intended to illustrate the hæmolytic, glossitic, gastric, and intestinal "infective" lesions of ganglionic or pernicious anæmia. The collection in the Barnfield Hall is further augmented by a large number of microscopic slides, for the inspection of which no less than 75 microscopes, lent by the Leitz Company, are available.

### The Exhibition.

In the Victoria Hall, Queen Street, is the exhibition of foods and drugs, surgical and other instruments and appliances. It is a somewhat disappointing collection; the exhibits are too crowded, and there is too little effective arrangement. The catalogue is excellent, but it is difficult to find the stalls. Want of space is doubtless one reason for the poor impression that the exhibition creates at first sight, and for that the management can scarcely be held responsible.

There is nothing that is strikingly new at the exhibition. The old makers exhibit their specialities, and in the department of instruments Messrs. Down Bros., The Holborn Surgical Instrument Company, Messrs. Arnold and Sons, Allen and Hanburys, Ltd., and Maw, Son, and Sons are prominent exhibitors. Among the stalls which should interest the general practitioner one may mention that of Meister, Lucius and Bruning, Ltd., who are showing samples of their tuberculins, and synthetic chemical preparations; and of the Miol Manufacturing Company, which has an attractive stand, and exhibits both the finished Miol and the constituents from which it is prepared. This food-preparation is likely to loom so large in the future that the opportunity of inspecting and trying it is not to be lost. The Denver Chemical Manufacturing Co., the Bayer Co., the Equipoise Couch Co., Knoll and Co., and the Marconi Co.'s stalls are also worth inspection. Of local firms there are several. H. Whiteway and Co., Ltd., of The Orchards, Whimple, Devon, have a stall devoted to cyders. The company's "Cydrax" is a pleasant non-intoxicating drink, and their perry, as sampled at the stall, is excellently dry. Another local firm is that of Evans, Gadd and Co., Ltd., of Exeter, to whose products stall 62 is devoted. This firm supplies various pharmaceutical preparations standardised by physical, chemical, and physiological tests. Their digitalis tincture is physiologically measured, and each bottle bears a special certificate warranting, as far as is possible, the efficaciousness of the preparation.

### The General Meeting.

At half past two on Tuesday afternoon members foregathered in the theatre near the Royal Assembly Rooms for the general meeting. This was the first opportunity of observing the association as a whole, and the first impression that the observer gained was that the members do not display much interest in the proceedings of the general meeting. There were scarcely five hundred ladies and gentlemen present, and it would be difficult to say how many of the latter were members. A posse of some thirty members of the executive occupied the stage; in the stalls were about forty more, but the greater part of the members seemed to prefer the dress circle.

The business of the afternoon was soon disposed of. The retiring President, Dr. Reeve, of Toronto, a kindly-faced, old-looking man with a pronounced American accent, made his bow and introduced the president-elect, Dr. Davy. The latter took the chair, after briefly having expressed his thanks to the Association for his election. Then came an unforeseen and pleasant little ceremony, the presenting of three copies of that excellent guide to Drs. Davy and Gordon and Mr. Russell Coombe, by the South Western Branch of the Association.

### Next Year's Meeting and the President-elect.

After that, Dr. J. A. MacDonald extended, on behalf of the Sheffield Branch, a cordial invitation to the Association to visit Sheffield next year. The invitation, which had been considered in council, was unanimously accepted, and Mr. Simeon Snell, F.R.C.S.Ed., was elected President-elect for the ensuing year. In thanking the Association for the honour done to him, Mr. Snell made an effective though necessarily brief speech, and detailed the attractions of Sheffield, promising the Association a most cordial welcome on behalf of his adopted city.

### The Budget.

Dr. Radcliffe Crocker, the retiring treasurer, moved the adoption of the financial statement. This was agreed to after a brief discussion. A member proposed the formation of a provident fund. Speaking with an earnestness and sincerity which evoked applause, he moved as a resolution that the Association should set aside a yearly sum of £1,000 to form the nucleus of such a fund. "I want," he said, "every member's wife to have the right to appeal to this Association when she is in want or distress." The chairman of the representative committee, who occupied the chair, put the proposal to the meeting with a curt and unsympathetic reminder that the Association had no power to deal with its funds in that manner, and the motion was lost. A voice from the dress circle asked the chairman to explain "to those who had not been present at the representative meeting" that the Association was not unfavourable to the establishment of a provident fund, and that the loss of the motion should "not be construed adversely."

### Cross Currents.

So far everything had proceeded smoothly. The Council had had it all its own way, and, "as agreed in council," members proposed, seconded, and sat down in monotonous and stereotyped fashion. Dr. Crocker regretfully intimated that he had to resign the treasurership, and the meeting regretfully accepted the resignation, and unanimously elected him a vice-president of the Association for life, an honour which had previously been accorded to the retiring president, Dr. Reeve. A member on the platform, at a nod from the chairman, next got up to propose as a candidate for the office of treasurer Mr. Charles R. Straton, F.R.C.S., a member of the representative meeting, and obviously the chosen of the Council. The proposition was duly seconded, but when the chairman rose to put the motion to the meeting, Sir Victor Horsley, who occupied a front seat in the dress circle, nominated a second candidate. Hitherto they had had treasurers from the south, he said, and he would propose a change to the north. His nomination was

Dr. Edwin Rayner, of Stockport (loud applause, such as even the votes of thanks had not called forth before), whom everyone knew as a capable financier. Sir Victor's speech was not gracefully received; the gentlemen on the platform made audible and disparaging remarks. For the moment the non-member, or the member who had not been privileged to swim in the currents that run in the representative and council meetings, felt a real note had been sounded. To the mass of members present, it was easy to see, Sir Victor's speech, carefully and diplomatically made as it was, came as an expression of general opinion. On the platform one heard mutterings of "Another opposition party"—in the stalls a gentleman rose and vehemently protested against "a slight to the Council," "a government by clique," but in the dress circle, where the majority of the members were seated, the Rayner party was undoubtedly predominant. Professor Morrison, of Birmingham, appealed from the back rows for unprejudiced consideration of the claims of Sir Victor's candidate, and asked members not to let any expression of opinion on medical or association politics stand in the way of electing a treasurer. The chairman appealed to members not to carry on the discussion, but to come to a vote at once, and as there was, apparently, no objection to that course, Dr. Rayner was declared duly elected, the show of hands having been greatly in his favour.

### The President's Address.

On Tuesday evening the President, Dr. Davy, delivered his inaugural address in the Theatre, which was fairly well filled for the occasion. Following the custom which has prevailed at such gatherings for the last 30 years, members wore academic or evening dress, and the effect of the various costumes with their multi-coloured hoods reminded one of a gala performance. On the platform were the members of the executive and the more prominent visitors, and prior to the delivery of his address the President received the foreign visitors and Colonial delegates, among the former being Professor Strassmann of Berlin, Professor Salomonsen of Amsterdam, Professor Fish of Chicago, and Professor Laudolt of Paris. After the reception the President presented the Middlemore Prize, consisting of a cheque for fifty guineas and an illuminated address, to Mr. Stephenson, the Middlemore prizeman for the year.

Dr. Davy then proceeded to read his address. He read it in a clear, strong voice that carried well, but possessed little modulation. It was not a commonplace address—it could hardly be that, giving many evidences of careful compilation and sincerity.

Dr. Davy commenced by shortly contrasting the condition of affairs, so far as medicine—or, as he preferred to say, the science of medicine—was concerned at the time of the last visit of the Association to Exeter in 1842 and at present. Some of his introductory remarks struck home. To a thoughtful audience—and a glance at the faces around him on the platform showed that there at least were men who followed his words with keen, meditative interest—they were weighty words. "A large part of the empiricism, of the empirical knowledge gained 40 years ago, is as good and as sound now as it was then," he remarked, and the words evoked a ripple of applause.

### Factors in Evolution.

Among the factors that had combined to advance medicine in its forward stride, Dr. Davy put the greater respect for scientific accuracy, the microscope, and the advances in chemistry first, but he went on to say that he thought the Darwinian theory had a great meaning for the profession. The work of Darwin had not been sufficiently highly regarded in medicine; it had served to stimulate, unnecessarily actively, discussion on purely academical questions, and the profession had lost sight of its importance on other points. The culture of health was the first necessity of life, and, dealing with this subject, the President embarked upon what seemed perilously like a tirade against modern civilisation. Environment—physical deterioration—machinery as a factor in muscular atrophy—these were his themes, and it seemed that he had his audience completely with him. "The working-man pays his few pence to see some experts at football or cricket." "A nation of muscular degenerates," a quotation from Kipling, evoked prolonged applause. He pointed out the importance of regulated exercise, of having such exercise judiciously superintended by qualified medical men. He went into the region of food reform, into medical inspection of schools, and pointed out what Japan had achieved.

### The Medical Aspect.

All this was old; it has been said over and over again, but coming from the President of the British Medical Association, and delivered with all the pomp that attaches to such ex cathedra statements, it will doubtless appeal to the public. To medical men the latter part of the address will prove much more interesting, for therein Dr. Davy dealt with tuberculosis as a factor in national deterioration. He took pyæmia and typhoid, and compared the results which had been achieved by a vigorous crusade against these pests with the little that has been done to exterminate consumption. Typhoid had practically disappeared. "My predecessor made £300 a year from typhoid cases . . . during the last few years my income from typhoid has hardly reached £5 a year," he said amidst a burst of laughter and applause. It was time that the medical profession spoke out on the question of tuberculosis; it was time that energetic efforts were made to deal with the scourge. He was fully aware of the difficulties that faced them in legislation, but he thought that it was their duty to deal with the matter, carefully and earnestly.

Professor Osler, in moving a vote of thanks, which was seconded by Sir Philip Jones, said he was grateful to Dr. Davy for having vigorously reminded them of their duties with regard to tuberculosis. The compulsory notification of tuberculosis was vexatious to some of them, but it was a question that had to be faced. He endorsed Dr. Davy's remarks concerning the necessity for a systematic medical inspection of schools, and for greater attention to the physical development of the school-going child.

